# Delivery of a one-week chewing gum for promoting smoking cessation: A pilot randomized controlled trial

**DOI:** 10.18332/tid/218790

**Published:** 2026-05-14

**Authors:** Meng Yao Li, Yin Ting Yiu, Sheng Zhi Zhao, Yee Tak Derek Cheung, Ke Deng, Tai Hing Lam, Man Ping Wang

**Affiliations:** 1 School of Nursing, Li Ka Shing Faculty of Medicine, The University of Hong Kong, Hong Kong, China; 2 School of Public Health, Li Ka Shing Faculty of Medicine, The University of Hong Kong, Hong Kong, China; 3 Periodontology and Implant Dentistry, Faculty of Dentistry, The University of Hong Kong, Hong Kong, China

**Keywords:** chewing gum, brief advice, smoking cessation, pilot projects

## Abstract

**INTRODUCTION:**

Chewing gum has been used as a self-help strategy for managing smoking cravings, yet its effectiveness has not been evaluated. This pilot trial assessed the feasibility, acceptability, and preliminary effectiveness of delivering 1-week chewing gum for promoting quitting.

**METHODS:**

Adult daily smokers were recruited from smoking hotspots in Hong Kong in February 2025. All participants received very brief advice (VBA) and a selfhelp booklet at baseline. The intervention group additionally received 1 week of chewing gum (2 packs) and 2 weeks of instant messaging reminders to promote gum usage, whereas the control group received no placebo or sham intervention beyond VBA and the booklet. Follow-up was conducted at 2 weeks, 3 months, and 6 months post-enrolment. The primary outcome was biochemically validated tobacco abstinence at 6 months. Risk ratios (RRs) were used to estimate the intervention effect using intention-to-treat analysis in Poisson regression.

**RESULTS:**

The recruitment rate was 91%, and the retention rate was 86%, 78%, and 74% at 2 weeks, 3 months, and 6 months, respectively. Eighty participants were randomly assigned to the intervention (n=40) and control (n=40) groups using a 1:1 allocation ratio. The intervention group had a higher validated abstinence (10% vs 0%; risk difference, RD=0.53; 95% CI: 0.41–0.64) at 6 months than the control group. Among intervention group participants, 75% (30/40) used chewing gum, and 57% (17/30) rated it as helpful in relieving cravings. Those who used the chewing gum had higher rates of quit attempts (77% vs 40%, p=0.03) and smoking reduction (73% vs 40%, p=0.06) than those who did not.

**CONCLUSIONS:**

Delivering chewing gum was feasible and showed preliminary evidence of effectiveness in cessation outcomes. Full trials with an extended intervention period are warranted.

**CLINICAL TRIAL REGISTRATION:**

The study is registered on the official website of ClinicalTrials.gov

**IDENTIFIER:**

CT06801860

## INTRODUCTION

Chewing gum has been used as an alternative to smoking cessation. Randomized trials have found chewing gum to be effective for reducing cravings and managing withdrawal symptoms^1-3^, primarily by substituting the habitual hand-to-mouth action associated with smoking^4^. Whether chewing gum, as a simple behavioral adjunct, can further improve smoking cessation outcomes remains inconclusive. A literature search of PubMed, the Cochrane Library, and ClinicalTrials.gov (up to 31 October 2025) using the terms ‘chewing gum’, ‘smoking cessation’, and ‘smoking reduction’ identified no trials evaluating the effectiveness of chewing gum on cessation outcomes. This pilot randomized controlled trial (RCT) assessed the feasibility, acceptability, and preliminary effectiveness of delivering a 1-week supply of chewing gum for quitting in daily smokers in Hong Kong.

## METHODS

### Study design and participant recruitment

We conducted a two-arm, parallel pilot RCT in Hong Kong between 11 February and 26 August 2025. Ethical approval was obtained from the Institutional Review Board of the University of Hong Kong/Hospital Authority Hong Kong West Cluster (UW24812), and the trial was registered with ClinicalTrials.gov (NCT06801860). Participants were recruited from community smoking hotspots, defined as public locations with a high concentration of smokers^5^. Trained research staff conducted on-site outreach by approaching adult smokers, briefly introducing the study, and screening interested individuals for eligibility. Eligible individuals were Hong Kong residents aged ≥18 years who smoked daily, spoke Cantonese, used instant messaging apps (e.g. WhatsApp), and were willing to use chewing gum. Exclusion criteria included communication barriers, contraindications to chewing gum (e.g. loose teeth, jaw joint disorders), or current engagement in other cessation programs. Participants received HK$100 (US$12.8) for completing the baseline assessment and HK$50 (US$6.4) for each follow-up. The study followed the CONSORT statement extension for pilot and feasibility trials^6^ (Supplementary file).

### Procedures

Participants were individually randomized to the intervention group or control group (at a 1:1 ratio) using a computer-generated, concealed allocation sequence with permuted block sizes of 2, 4, and 6. Masking of participants and research staff was not feasible because of the nature of the intervention. Outcome assessors and statistical analysts remained masked until completion of the primary analyses.

At baseline, all participants received very brief advice (VBA) following the AWARD model and a 12-page self-help booklet outlining strategies to manage withdrawal symptoms. Participants in the intervention group additionally received a 2-week support consisting of a 1-week supply of chewing gum (Extra^®^; 2 packs; US$1.5 per pack) provided in person at baseline recruitment, and 2 weeks of instant messaging reminders (twice per week, 4 in total). The reminders grounded in the Love and Care approach, provided brief and empathetic encouragement without behavioral counselling and functioned only as prompts to remind gum adherence. A leaflet summarized the potential role of chewing gum in supporting smoking cessation and oral health, and the proper gum usage, recommended timing, dosage, and disposal were provided to the participants. Participants were encouraged to use the gum when experiencing smoking urges. Chewing gum is generally safe, although participants were advised to report any allergies or adverse effects. Participants in the control group only received VBA at baseline.

### Outcomes

Data were collected by questionnaire at baseline and telephone follow-up at 2 weeks, 3 months, and 6 months, after intervention initiation. The primary outcome was biochemically validated past 7-day reported tobacco abstinence at 6 months (exhaled CO <4 ppm measured by a Smoklyzer, and salivary cotinine <10 ng/mL measured by NicAlert^®^ strips)^7^. Secondary outcomes included self-reported 7-day point-prevalence abstinence (PPA), ≥50% reduction in daily cigarette consumption (defined by a reduction of at least 50% in daily cigarette consumption compared with that at baseline; excluding quitters), and quit attempts (≥24 hours of abstinence) at 2 weeks, 3 months, and 6 months follow-up. Feasibility outcomes included recruitment rate, retention rate at each follow-up, and adherence to the intervention, defined as the proportion of participants who used the xylitol gum as instructed. Acceptability was evaluated through user ratings of perceived helpfulness of the gum for smoking reduction and cessation (0–10 scale, higher score indicating greater perceived helpfulness) and perceived oral-health benefits (4 items rated on a 0–5 scale, higher score indicating greater perceived helpfulness). Participants received HK$250 (US$32) for each successful validation at 3 months and 6 months.

### Statistical analysis

No formal sample-size calculation was performed for the pilot RCT. Our previous pilot RCT on smoking cessation (n=80) was considered adequate for feasibility assessment and estimation of parameters for a future definitive trial^8^.

Descriptive statistics were used to summarize participant characteristics and cessation outcomes. Continuous variables are presented as means and standard deviations (SD), and categorical variables as frequencies and percentages. Primary analyses were by intention-to-treat (ITT), assuming no behavior change for participants lost to follow-up^9^. Group differences were examined using Poisson regression with robust variance to estimate risk ratios^10^, risk differences were reported when zero events occurred. Quitting outcomes were analyzed using both unadjusted and adjusted models, with the latter adjusted for key predictors of cessation outcomes, including previous quit attempts, intention to quit, and nicotine dependence^11^. The smoking reduction by at least 50% was analyzed, excluding participants who self-reported quitting. We did a planned sensitivity analysis using complete-case analysis. *Post hoc* as-treated analysis was conducted to evaluate outcomes based on the intervention actually received rather than the initial assignment. All analyses were performed in Stata/MP 15.1 (StataCorp), with two-sided p<0.05 considered statistically significant.

## RESULTS

In February 2025, 88 individuals were screened, of whom 80 were enrolled, resulting in a 91% recruitment rate. Forty participants were randomized to the intervention and 40 to the control group (Supplementary file Figure 1). Most participants were male (64/80; 80%), 70% (56/80) were aged <50 years, and 73% (58/80) had a tertiary education. At baseline, 51% (41/80) had moderate to high nicotine dependence, and 71% (57/80) had no plan to quit within 30 days. Baseline characteristics were similar between groups (Supplementary file Table 1). Follow-up retention was 86% at 2 weeks (n=69), 78% at 3 months (n=62), and 74% at 6 months (n=59), corresponding to 11, 18, and 21 participants with missing outcome at each time point. Retention did not differ by group (retention rate ratio ranged from 1.03 to 1.21).

Table 1 shows that based on ITT analysis, validated tobacco abstinence was higher in the intervention group than in the control group at 6 months (4/40; 10% vs 0/40; 0%; RD=0.53; 95% CI: 0.41–0.64) but similar at 3 months (2/40; 5% vs 2/40; 5%; RR=1.00; 95% CI: 0.15–6.84). Self-reported 7-day PPA was similar at 6 months (20% vs 18%; RR=1.14; 95% CI: 0.46–2.87) but lower in the intervention than the control group at 3 months (10% vs 15%; RR=0.67; 95% CI: 0.20–2.20) and 2 weeks (0% vs 13%; RR= -0.53; 95% CI: -0.65 – -0.42). More participants in the intervention group reported smoking reduction (excluding quitters; RR: 1.52–1.91) and quit attempts (RR: 1.13–1.30) across follow-ups, except for smoking reduction at 6 months (13% vs 18%; RR=0.69; 95% CI: 0.21–2.23). Sensitivity analyses using complete-case and as-treated approaches yielded consistent results (Supplementary file Table 2).

**Table 1:** Smoking cessation outcomes at multiple follow-up time points comparing intervention versus control groups in a 2-arm pilot randomized controlled trial in Hong Kong, 11 February – 26 August 2025 (intention-to-treat analysis; N=80) ^a^

	*Intervention* *(N=40) n (%)*	*Control (N=40)* *n (%)*	*Crude risk ratio (RR)/* *Crude risk difference* *(RD) (95% CI)*	*p*
**Validated abstinence**				
3 months	2 (5)	2 (5)	1.00 (0.15–6.84)	1.0
6 months (primary outcome)^b^	4 (10)	0 (0)	0.53 (0.41–0.64)	0.04
**Self-reported PPA** ^c^				
2 weeks^b^	0 (0)	5 (13)	-0.53 (-0.65 – -0.42)	0.02
3 months	4 (10)	6 (15)	0.67 (0.20–2.20)	0.51
6 months	8 (20)	7 (18)	1.14 (0.46–0.87)	0.78
**Smoking reduction** ^d^				
2 weeks	26 (65)	13 (37)	1.75 (1.07–2.86)	0.03
3 months	8 (23)	6 (18)	1.30 (0.50–3.36)	0.60
6 months	4 (13)	6 (18)	0.69 (0.21–2.23)	0.53
**Quitting attempts**				
2 weeks	27 (68)	21 (53)	1.29 (0.89–1.86)	0.18
3 months	13 (33)	10 (25)	1.30 (0.64–2.62)	0.46
6 months	18 (45)	16 (40)	1.13 (0.67–1.88)	0.65

a Missing observations were treated as not quitting or reducing.

b Risk difference was reported only for 6-month biochemically validated abstinence and 2-week self-reported 7-day PPA because the risk ratio could not be estimated due to complete separation (i.e. zero events in one group).

c PPA: point-prevalence abstinence.

d At least a 50% reduction in baseline daily cigarette consumption; participants who self-reported quitting were excluded.

In the intervention group, 75% (30/40) of participants used chewing gum in the past 7 days, and 60% (18/30) users reported using it on all 7 days (Supplementary file Table 3). Among gum-users, 57% (17/30) reported that the gum helped reduce smoking cravings, and the overall rating for its usefulness in smoking reduction and cessation was 4.10 (SD=2.77). Participants also reported perceived benefits in relieving oral dryness (mean=2.67, SD=1.71), refreshing breath (mean=3.13, SD=1.61), increasing saliva secretion (mean=2.63, SD=1.61), and reducing oral discomfort (mean=2.20, SD=1.54). No adverse events were reported by participants.

*Post hoc* analysis showed that participants who used the gum at least one day had higher rates of 2-week smoking reduction (73% vs 40%) and 2-week quit attempts (77% vs 40%) compared with non-users (Supplementary file Table 4).

## DISCUSSION

This pilot RCT evaluated the delivery of 1-week chewing gum for quitting in community smokers, showing that chewing gum was acceptable and provided preliminary evidence of effectiveness, with a higher biochemically validated abstinence rate at 6 months. Chewing gum may be considered a low-threshold option for smokers who are unwilling or reluctant to use nicotine replacement therapy (NRT) or other pharmacological cessation aids. Our findings support the need for a larger trial to explore how offering chewing gum increases quit attempts and sustained abstinence.

Despite the low willingness to quit at baseline, most participants in the intervention group used the chewing gum. The usage rate in this pilot trial was higher than that reported in our previous RCTs offering 1-week NRT among smokers with low quit intention^12^, and among daily smokers recruited from smoking hotspots^13^. This might be due to the ease of use, low cost, and wide accessibility of chewing gum, which make it suitable for smokers who are not ready to quit. Given its non-pharmacological nature and the absence of medication-related side effects^14^, chewing gum could serve as a supplementary option within smoking cessation services, especially for individuals who are not suited to NRT in future applications.

In this trial, more than half of the participants using chewing gum reported that it helped reduce smoking cravings, which may be due to the act of chewing occupying the mouth, distracting attention from cravings, and providing psychological relief for smokers^15^. These findings were consistent with RCTs showing that chewing gum can assist in managing smoking cravings, particularly in smoking-restricted settings^1,2^. Our results further showed that higher proportions of gum users reported smoking reduction and quit attempts than non-users at 2 weeks post-enrolment. Future studies should explore the optimal duration and dosage of gum use (e.g. extending provision beyond 2 weeks), identify mHealth-based strategies to improve adherence, and combine with other behavioral cessation interventions (e.g. motivational interviewing, chat-based instant messaging) to enhance cessation support.

### Limitations

Our study had limitations. The intervention period was brief, which restricted our ability to assess the durability of behavioral change. The short exposure may limit the examination of dose-response patterns and long-term adherence. The abstinence rate observed in this study was lower than that reported in previous trials using NRT gum at 6 months^16^. However, this comparison should be interpreted cautiously because the intervention was not designed to replace pharmacotherapy, and the study was not powered to evaluate effectiveness. The secondary outcomes of intervention engagement (e.g. perceived helpfulness of the gum for smoking reduction and cessation, and perceived oral-health benefits) were based on self-reports. We did not assess oral-health improvement, and this outcome will be evaluated in a separate, dedicated trial. The reminder messages were included solely to prompt gum use and did not contain cessation counselling, making it unlikely that the outcomes were driven by the reminders. Our pilot trial was conducted in Hong Kong, where the prevalence of smoking is low, and smokers are predominantly male^17^. The generalizability of the findings to other places is unclear.

## CONCLUSIONS

We showed the initial feasibility and acceptability of delivering 1-week chewing gum for smoking cessation and preliminary evidence of its potential effectiveness. Large trials with extended intervention periods are warranted to evaluate its effectiveness more definitively.

## Supplementary Material



## Data Availability

The data supporting this research are available from the authors on reasonable request.

## References

[1] Cortez-Garland M, Cohen LM, Vanderveen JW, Cook K. The effect of chewing gum on self-reported nicotine withdrawal: Is it the flavor, the act of chewing, or both?. Addict Behav. 2010;35(3):224-228. doi:10.1016/j.addbeh.2009.10.016 19914004

[2] Cohen LM, Collins FL Jr, Vanderveen JW, Weaver CC. The effect of chewing gum flavor on the negative affect associated with tobacco abstinence among dependent cigarette smokers. Addict Behav. 2010;35(11):955-960. doi:10.1016/j.addbeh.2010.06.010 20598808

[3] Cohen LM, Britt DM, Collins FL, Al’Absi M, McChargue DE. Multimodal assessment of the effect of chewing gum on nicotine withdrawal. Addict Behav. 2001;26(2):289-295. doi:10.1016/s0306-4603(00)00100-3 11316385

[4] Cohen LM, Britt DM, Collins FL Jr, Stott HD, Carter LC. Chewing gum affects smoking topography. Exp Clin Psychopharmacol. 1999;7(4):444-447. doi:10.1037//1064-1297.7.4.444 10609978

[5] Cheung YTD, Lam TH, Li WHC, Wang MP, Chan SSC. Feasibility, efficacy, and cost analysis of promoting smoking cessation at outdoor smoking “hotspots”: A pre-post study. Nicotine Tob Res. 2018;20(12):1519-1524. doi:10.1093/ntr/ntx147 28655173

[6] Eldridge SM, Chan CL, Campbell MJ, et al. CONSORT 2010 statement: Extension to randomised pilot and feasibility trials. BMJ. 2016;355:i5239. doi:10.1136/bmj.i5239 27777223 PMC5076380

[7] Piper ME, Bullen C, Krishnan-Sarin S, et al. Defining and measuring abstinence in clinical trials of smoking cessation interventions: An updated review. Nicotine Tob Res. 2020;22(7):1098-1106. doi:10.1093/ntr/ntz110 31271211 PMC9633719

[8] Guo Z, Wu Y, Wang MP. Mobile phone–based personalized and interactive augmented reality pictorial health warnings for enhancing a brief advice model for smoking cessation: Pilot randomized controlled trial. JMIR XR and Spatial Computing (JMXR). 2024;1(1):e52893. doi:10.2196/52893 PMC13179107 42147208

[9] West R, Hajek P, Stead L, Stapleton J. Outcome criteria in smoking cessation trials: Proposal for a common standard. Addiction. 2005;100(3):299-303. doi:10.1111/j.1360-0443.2004.00995.x 15733243

[10] Zou G. A modified poisson regression approach to prospective studies with binary data. Am J Epidemiol. 2004;159(7):702-706. doi:10.1093/aje/kwh090 15033648

[11] Vangeli E, Stapleton J, Smit ES, Borland R, West R. Predictors of attempts to stop smoking and their success in adult general population samples: A systematic review. Addiction. 2011;106(12):2110-2121. doi:10.1111/j.1360-0443.2011.03565.x 21752135

[12] Luk TT, Lam TH, Leung WC, et al. Brief advice, nicotine replacement therapy sampling, and active referral for expectant fathers who smoke cigarettes: A randomized clinical trial. JAMA Intern Med. 2021;181(8):1081-1089. doi:10.1001/jamainternmed.2021.2757 34125135 PMC8204256

[13] He WJA, Wang Q, Chan CHH, et al. Effectiveness of mobile smoking cessation treatment with 1-week nicotine replacement therapy sampling at outdoor smoking hotspots: A cluster randomized controlled trial. Addiction. 2025;120(1):106-115. doi:10.1111/add.16666 39256314 PMC11638493

[14] Allam A, Cirio S, Salerno C, Camoni N, Campus G, Cagetti MG. Chewing gum and health: A mapping review and an interactive evidence gap map. Nutrients. 2025;17(17):2749. doi:10.3390/nu17172749 40944140 PMC12430410

[15] Britt DM, Cohen LM, Collins FL Jr, Cohen ML. Cigarette smoking and chewing gum: Response to a laboratory-induced stressor. Health Psychol. 2001;20(5):361-368. doi:10.1037//0278-6133.20.5.361 11570650

[16] Hartmann-Boyce J, Chepkin SC, Ye W, Bullen C, Lancaster T. Nicotine replacement therapy versus control for smoking cessation. Cochrane Database Syst Rev. 2018;5(5):CD000146. doi:10.1002/14651858.CD000146.pub5 29852054 PMC6353172

[17] Census and Statistics Department. Thematic Household Survey Report - Report No. 79 Pattern of Smoking. Accessed March 6, 2026. https://www.censtatd.gov.hk/en/wbr.html?ecode=B11302012024XX01

