# Supplementary material for: Delivery of a one-week chewing gum for promoting smoking cessation: A pilot randomized controlled trial
Source: Tob Induc Dis. 2026 May 14;24:10.18332/tid/218790. doi: 10.18332/tid/218790 (PMC13175150; doi:10.18332/tid/218790)
Supplement: Supplementary file 1 [file TID-24-61-s1.pdf]

CONSORT 2010 checklist of information to include when reporting a pilot or feasibility trial*

Section/Topic	Item No	Checklist item	Reported on page No
**Title and abstract**			
	1a	Identification as a pilot or feasibility randomised trial in the title	P1
	1b	Structured summary of pilot trial design, methods, results, and conclusions (for specific guidance see CONSORT abstract extension for pilot trials)	P2
**Introduction**			
Background and objectives	2a	Scientific background and explanation of rationale for future definitive trial, and reasons for randomised pilot trial	P3
	2b	Specific objectives or research questions for pilot trial	P3
**Methods**			
Trial design	3a	Description of pilot trial design (such as parallel, factorial) including allocation ratio	P3
	3b	Important changes to methods after pilot trial commencement (such as eligibility criteria), with reasons	NA
Participants	4a	Eligibility criteria for participants	P3
	4b	Settings and locations where the data were collected	P3
	4c	How participants were identified and consented	P3
Interventions	5	The interventions for each group with sufficient details to allow replication, including how and when they were actually administered	P4
Outcomes	6a	Completely defined prespecified assessments or measurements to address each pilot trial objective specified in 2b, including how and when they were assessed	P4-5
	6b	Any changes to pilot trial assessments or measurements after the pilot trial commenced, with reasons	NA
	6c	If applicable, prespecified criteria used to judge whether, or how, to proceed with future definitive trial	P5
Sample size	7a	Rationale for numbers in the pilot trial	P4
	7b	When applicable, explanation of any interim analyses and stopping guidelines	NA
Randomisation:			
Sequence generation	8a	Method used to generate the random allocation sequence	P3
	8b	Type of randomisation(s); details of any restriction (such as blocking and block size)	P3
Allocation concealment mechanism	9	Mechanism used to implement the random allocation sequence (such as sequentially numbered containers), describing any steps taken to conceal the sequence until interventions were assigned	P3

Implementation	10	Who generated the random allocation sequence, who enrolled participants, and who assigned participants to interventions	P3
Blinding	11a	If done, who was blinded after assignment to interventions (for example, participants, care providers, those assessing outcomes) and how	P4
	11b	If relevant, description of the similarity of interventions	P4
Statistical methods	12	Methods used to address each pilot trial objective whether qualitative or quantitative	P4
**Results**			
Participant flow (a diagram is strongly recommended)	13a	For each group, the numbers of participants who were approached and/or assessed for eligibility, randomly assigned, received intended treatment, and were assessed for each objective	P5
	13b	For each group, losses and exclusions after randomisation, together with reasons	Supplemental Figure 1
Recruitment	14a	Dates defining the periods of recruitment and follow-up	P5
	14b	Why the pilot trial ended or was stopped	NA
Baseline data	15	A table showing baseline demographic and clinical characteristics for each group	Supplemental Table 1
Numbers analysed	16	For each objective, number of participants (denominator) included in each analysis. If relevant, these numbers should be by randomised group	Table 1
Outcomes and estimation	17	For each objective, results including expressions of uncertainty (such as 95% confidence interval) for any estimates. If relevant, these results should be by randomised group	Table 1
Ancillary analyses	18	Results of any other analyses performed that could be used to inform the future definitive trial	Supplemental Table 2
Harms	19	All important harms or unintended effects in each group (for specific guidance see CONSORT for harms)	Supplemental Table 3
	19a	If relevant, other important unintended consequences	NA
**Discussion**			
Limitations	20	Pilot trial limitations, addressing sources of potential bias and remaining uncertainty about feasibility	P7
Generalisability	21	Generalisability (applicability) of pilot trial methods and findings to future definitive trial and other studies	P6
Interpretation	22	Interpretation consistent with pilot trial objectives and findings, balancing potential benefits and harms, and considering other relevant evidence	P6
	22a	Implications for progression from pilot to future definitive trial, including any proposed amendments	P6
**Other information**			
Registration	23	Registration number for pilot trial and name of trial registry	P1 and P3
Protocol	24	Where the pilot trial protocol can be accessed, if available	P1 and P3

Funding	25	Sources of funding and other support (such as supply of drugs), role of funders	P1
	26	Ethical approval or approval by research review committee, confirmed with reference number	P3

Citation: Eldridge SM, Chan CL, Campbell MJ, Bond CM, Hopewell S, Thabane L, et al. CONSORT 2010 statement: extension to randomised pilot and feasibility trials. BMJ. 2016;355. This is an Open Access article distributed in accordance with the terms of the Creative Commons Attribution (CC BY 3.0) license (http://creativecommons.org/licenses/by/3.0/), which permits others to distribute, remix, adapt and build upon this work, for commercial use, provided the original work is properly cited.

*We strongly recommend reading this statement in conjunction with the CONSORT 2010, extension to randomised pilot and feasibility trials, Explanation and Elaboration for important clarifications on all the items. If relevant, we also recommend reading CONSORT extensions for cluster randomised trials, non-inferiority and equivalence trials, non-pharmacological treatments, herbal interventions, and pragmatic trials. Additional extensions are forthcoming: for those and for up-to-date references relevant to this checklist, see www.consort-statement.org.

Supplemental Figure 1. Trial flow chart for the 2-arm pilot randomized controlled trial conducted in Hong Kong between 11 February and 26 August 2025

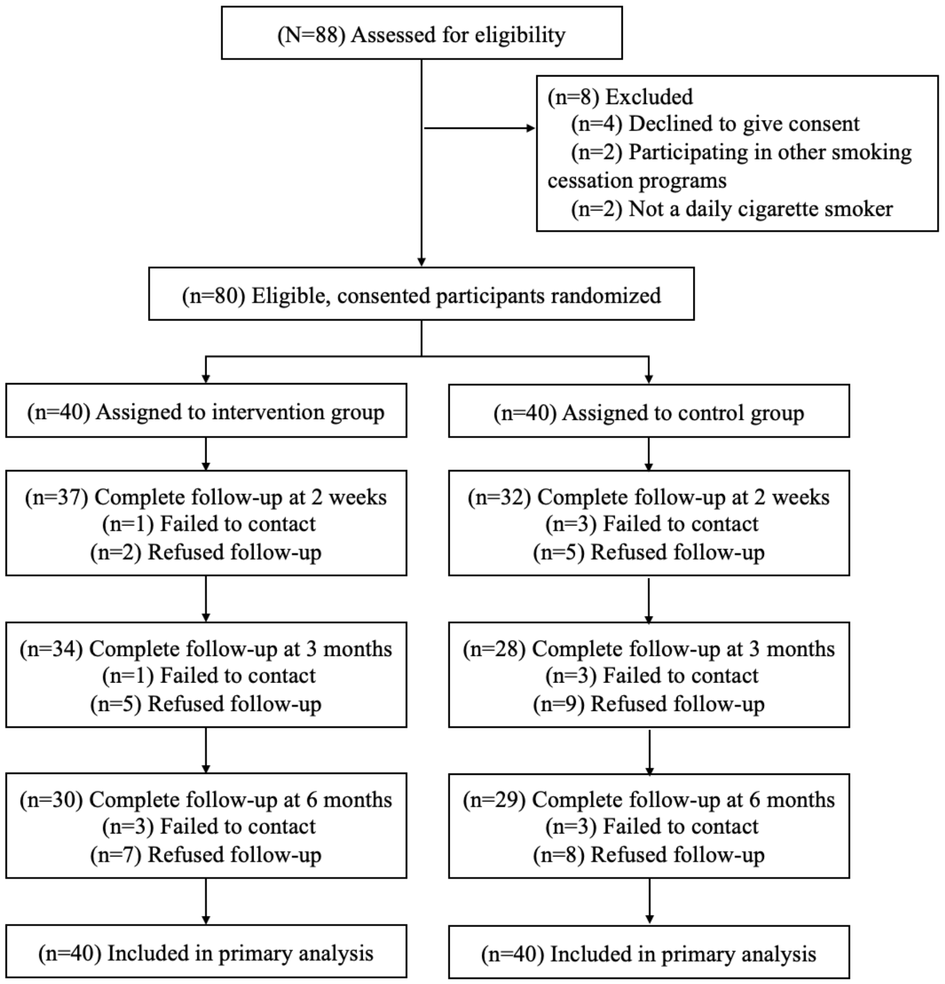

Supplemental Table 1. Baseline participants' characteristics for the 2-arm pilot randomized controlled trial conducted in Hong Kong between 11 February and 26 August 2025 (N=80)

Characteristics	Intervention group (n=40)	Control group (n=40)	*P* values [a]
**Sex, n (%)**			0.58
Male	31 (78)	33 (83)	
Female	9 (23)	7 (18)	
**Age (years), n (%)**			0.24
18-29	11 (28)	12 (30)	
30-39	10 (25)	9 (23)	
40-49	4 (10)	10 (25)	
≥50	15 (38)	9 (23)	
Mean (SD)	41.58 (16.06)	40.73 (16.69)	0.82
**Education, n (%)**			0.32
Secondary or below	13 (33)	9 (23)	
Tertiary	27 (68)	31 (78)	
**Monthly household income (US $1=HK $7.8), n (%)**			
<HK$25,000	13 (33)	14 (35)	0.97
HK$25,000-$60,000	19 (48)	18 (45)	
>HK$60,000	8 (20)	8 (20)	
**Employment, n (%)** [b]			0.59
Economically inactive	10 (25)	8 (20)	
Economically active	30 (75)	32 (80)	
**Marital status, n (%)**			0.72
Single	20 (50)	23 (58)	
Married	18 (45)	16 (40)	
Divorced or widowed	2 (5)	1 (3)	
**Living with a child younger than 18 years, n (%)**			0.58
No	33 (83)	31 (78)	
Yes	7 (18)	9 (23)	
**Daily cigarette consumption, mean (SD)**	12.71 (7.66)	12.86 (7.31)	0.93
**Nicotine dependency level, n (%)** [c]			0.81
Light	17 (45)	19 (49)	
Moderate	19 (50)	19 (49)	
Heavy	2 (5)	1 (3)	
**Past quit attempt, n (%)**			0.50
Within past 1 month	1 (3)	2 (5)	
Within past 6 months	2 (5)	3 (8)	
Within past 1 year	3 (8)	4 (10)	
More than 1 year	27 (68)	19 (48)	
Never	7 (18)	12 (30)	
**Willing to quit, n (%)**			0.50
Within 7 days	11 (28)	10 (25)	
Within 30 days	0 (0)	2 (5)	
Within 60 days	2 (5)	1 (3)	
Undetermined	27 (68)	27 (68)	
**Self-efficacy, mean (SD)** [d]			
Perceived confidence of quitting	4.98 (3.22)	5.23 (3.08)	0.72
Perceived difficulty of quitting	6.73 (3.03)	6.55 (3.17)	0.80
Perceived importance of quitting	5.40 (3.38)	5.95 (3.29)	0.46

Note: SD-Standard Deviations

[a] *P* values were calculated with the x^2 test or Fish exact test for categorical variables and a *t* test for continuous variables.

[b] Economically active: employed or self-employed; economically inactive: student, housekeeper, retired, or unemployed.

[c] Measured by the Heaviness of Smoking Index (HSI): HSI score≤2=light; HSI score 3-4=moderate; HSI score 5-6=heavy.

[d] Score range 0-10; higher score indicate higher perceived importance, confidence, and difficulty of quitting.

Supplemental Table 2. Sensitivity analysis of smoking cessation outcomes of the 2-arm pilot randomized controlled trial conducted in Hong Kong between 11 February and 26 August 2025 (N=80) [a]

	**Adjusted model** [b]		**Complete-case analysis**		**As-treated analysis**	
	**RR/RD(95% CI)**	***P* value**	**RR/RD(95% CI)**	***P* value**	**RR (95% CI)**	***P* value**
**Validated abstinence**						
3 months	0.66 (0.07, 5.91)	0.71	0.82 (0.12, 5.56)	0.84	0.47 (0.05, 4.43)	0.51
6 months [c]	0.09 (-0.01, 0.18)	0.08	0.53 (0.40, 0.66)	0.04	1.42 (0.21, 9.72)	0.72
**Self-reported PPA** [d]						
2 weeks [c]	-0.13 (-0.24, -0.03)	0.01	-0.58 (-0.70, -0.46)	0.01	0.36 (0.04, 3.08)	0.35
3 months	0.48 (0.10, 2.28)	0.36	0.55 (0.17, 1.77)	0.32	0.81 (0.26, 2.58)	0.73
6 months	0.92 (0.34, 2.47)	0.87	1.10 (0.46, 2.67)	0.83	0.71 (0.27, 1.90)	0.50
**Smoking reduction** [e]						
2 weeks	1.91 (1.10, 3.32)	0.02	1.35 (0.88, 2.09)	0.17	1.93 (1.23, 3.02)	0.004
3 months	1.52 (0.51, 4.53)	0.45	1.01 (0.41, 2.51)	0.98	2.48 (0.92, 6.68)	0.07
6 months	1.17 (0.30, 4.51)	0.82	0.64 (0.21, 1.97)	0.43	1.98 (0.61, 6.42)	0.25
**Quitting attempts**						
2 weeks	1.24 (0.84, 1.82)	0.28	1.11 (0.81, 1.53)	0.52	1.68 (1.18, 2.40)	0.004
3 months	1.18 (0.58, 2.42)	0.64	1.07 (0.55, 2.07)	0.84	1.85 (0.92, 3.72)	0.08
6 months	1.15 (0.67, 1.99)	0.61	1.09 (0.70, 1.69)	0.71	1.42 (0.86, 2.36)	0.17

Note: CI-Confidence Interval

[a] Missing observations were treated as not quitting or reducing

[b] Adjusted for past quit attempts, willing to quit, and nicotine dependence.

[c] Risk difference (RD) was reported only for 6-month biochemically validated abstinence and 2-week self-reported 7-day PPA because the Risk ratio (RR) could not be estimated due to complete separation (i.e., zero events in one group).

[d] PPA: point-prevalence abstinence.

[e] At least a 50% reduction in baseline daily cigarette consumption; participants who self-reported quitting were excluded.

Supplemental Table 3. Using chewing gum during the intervention period and related perceptions in the 2-arm pilot randomized controlled trial in Hong Kong between 11 February and 26 August 2025 (n=40).

	Values
**Using chewing gum, n (%)**	
Used	30 (75)
Never used [a]	10 (25)
**Number of usage days within users**	
1-3	8 (27)
4-6	4 (13)
7	18 (60)
**Among gum-users, "Chewing gum can help to..." (rating of perceptions), mean (SD)** [b]	
Relieve oral dryness	2.67 (1.71)
Refresh breath	3.13 (1.61)
Increase saliva secretion	2.63 (1.61)
Reduce oral discomfort	2.20 (1.54)
**Among gum-users, "After using chewing gum, it can help to...", n (%)** [c]	
Reduce the craving for smoking	17 (57)
Relieve anxiety after quitting smoking	4 (13)
Increase confidence in quitting smoking	7 (23)
Did not cause any noticeable change	14 (47)
**Perceived helpfulness in the process of smoking reduction or cessation among gum-users, mean (SD)** [d]	4.10 (2.77)
**Adverse event, n (%)**	
Yes	0 (0)
No	30 (100)

Note: SD-Standard Deviations

[a] Missing observations were treated as never used chewing gum.

[b] Scores ranged from 0 to 5, with higher scores indicating greater perceived helpfulness by participants.

[c] Multiple choice were allowed.

[d] Scores ranged from 0 to 10, with higher scores indicating greater perceived helpfulness by participants.

Supplemental Table 4. Smoking cessation outcomes at 2 weeks by chewing gum use in the intervention group of the 2-arm pilot randomized controlled trial in Hong Kong between 11 February and 26 August 2025 (n=40) [a]

	**Never used chewing gum (n=10), n (%)**	**Used chewing gum (n=30), n (%)**	***P* value**
Self-reported PPA [b]	0 (0)	0 (0)	NA
Smoking reduction [c]	4 (40)	22 (73)	0.06
Quit attempt	4 (40)	23 (77)	0.03

Note: NA-Not Applicable

[a] Missing observations were treated as never used chewing gum.

[b] PPA: point-prevalence abstinence.

[c] At least a 50% reduction in baseline daily cigarette consumption; participants who self-reported quitting were excluded.

© 2026 Li M.Y. et al.
